# A water additive with pomegranate can reduce dental plaque and calculus accumulation in dogs

**DOI:** 10.3389/fvets.2023.1241197

**Published:** 2023-09-29

**Authors:** Jerzy P. Gawor, Daria Ziemann, Celine S. Nicolas

**Affiliations:** ^1^Klinika Weterynaryjna Arka, Krakow, Poland; ^2^Virbac SA, GMMD Department, Carros, France

**Keywords:** dental plaque dog, Aquadent efficacy, limiting dental deposits, reducing gingivitis, periodontal disease prevention, dental homecare, pomegranate efficacy

## Abstract

Oral homecare plays a major part in dental disease prevention but it can be difficult to perform and time-consuming. Furthermore, the product used can be of limited efficiency. The goal of this study was to assess the efficacy of a water additive to limit the accumulation of plaque and calculus in dogs. Forty dogs were selected and randomly allocated to one of the two groups after scaling and polishing on day 0. The control group received no oral hygiene while the second group received the water additive (Vet Aquadent^®^ FR3SH^™^, Virbac) every day. After 30 days, plaque and calculus accumulations were evaluated under anesthesia. The Gingival Bleeding Index (GBI) was assessed on days 0 and 30. On day 30, the plaque and calculus indices were significantly smaller (*p* < 0.05) in the Aquadent group compared to the control group with median (Q1-Q3) scores of 1.22 (0.99–1.44) *vs*. 2.31 (1.65–3.86), respectively for plaque and 0.25 (0.15–0.42) *vs*. 0.33 (0.32–0.69) for calculus. Between day 0 and day 30, the GBI significantly decreased in the control group [from 0.39 (0.21–0.56) to 0.19 (0.08–0.29)] and in the Aquadent group [from 0.33 (0.18–0.47) to 0.00 (0.00–0.00)] but the decrease was significantly greater in the Aquadent group. These results show for the first time that the water additive tested can reduce dental deposit accumulation in dogs and improve gingival health. It can be recommended after a dental cleaning, especially to owners who are reluctant to provide dental care at home due to a lack of time or convenience.

## 1. Introduction

Periodontal disease was found to be the most prevalent disorder diagnosed in primary veterinary care in a recent study (1). Indeed, the incidence of such disease could reach 90% of all small animal patients (2). Although it can be present in young dogs, the incidence of the disease increases with age and in certain dog breeds like in toy and small breed dogs. Such a high prevalence can be due to a lack of education and knowledge but also to the fact that the disease can be kept unnoticed for a long time due to a lack of outward clinical signs (2). Preventing the appearance of such disease is the responsibility of the veterinarian but also of the owners who can help by providing dental care at home. In fact, a regular professional teeth cleaning is necessary but not sufficient to keep the teeth clean and the gingiva healthy as the plaque and calculus can form in a few days and gingivitis in a couple of weeks (2). Daily tooth brushing remains the gold standard for dental homecare but it is not easily performed by owners and not accepted by every dog. It is also time-consuming and therefore hardly implemented (3). Even when recommended by a veterinarian with all information given, compliance remains low (4). Alternatives to daily tooth brushing include diets, dental chews, and water additives. Several products are now available on the market but very few have proven their efficacy against plaque and calculus in controlled studies. Tooth brushing, kibbles, and dental chews can help control the accumulation of plaque and calculus thanks to their mechanical actions but water additives do not have such properties. Their action is mainly based on the chemical efficacy of the ingredients it is composed of. Vet Aquadent® FR3SH™ (Virbac) is a water additive containing three main ingredients of natural origin: pomegranate, erythritol, and inulin. These ingredients have demonstrated a certain efficacy to control bacterial growth and oral malodor, but mainly in humans or *in vitro* (5–11). In a recent *in vitro* study, the pomegranate extract present in Vet Aquadent® FR3SH™ water additive was found to be effective to limit the growth of canine oral bacterial species such as *Neisseria canis* or *Porphyromonas gulae*, even when organized in biofilms (12). These species are involved in plaque formation and periodontal disease progression (13–16). Limiting their growth could therefore help control the accumulation of dental plaque and calculus and the appearance and progression of periodontal disease in dogs.

The goal of this study was to assess the efficacy of the water additive containing the aforementioned pomegranate extract (Vet Aquadent^®^ FR3SH^™^) to control plaque and calculus accumulation and to improve gingival health in dogs, after a professional scaling.

## 2. Method

### 2.1. Animals

The 40 dogs included in the study were client-owned and all procedures performed during the study were conducted according to standard veterinary care procedures and following Polish Regulations (Art.1 ust.2 pkt1 Dz. U.2015 poz. 266). The study was approved by an ethical committee (Approbation numbers: EU-ERC/202205–01).

To be included, dogs of any body weight had to be in good general health and have mild to moderate gingivitis (Gingivitis index ≤2). All teeth to be scored (maxilla: I3, C, P3, P4, Ml, and mandible: C, P3, P4, Ml) had to be present as advised by the Veterinary Oral Health Council (see: VOHC.org), with no sign of periodontal disease exceeding the second stage (PD2), according to the American Veterinary Dental College (AVDC) staging (17). An oral examination was first performed on awake animals to qualify dogs for the trial based on the presence of required teeth and absence of obvious signs of advanced periodontal diseases. The inclusion criteria were assessed again on Day 0 on anesthetized animals and dogs exceeding PD2 stage were excluded. The researchers originally recruited more dogs than needed for the study to take into account the likelihood of excluding dogs with a more advanced stage of periodontal disease than PD2.

Dogs enrolled in the study belonged to 14 different breeds or mixed breeds, including small breeds (like Yorkshire terriers or Jack Russell terriers) and larger breeds (like German shepherds and Alaskan huskies).

All animals participating underwent qualification for anesthesia including a physical examination as well as blood and urine evaluation. If qualified, the dogs were anesthetized and a professional teeth cleaning procedure was performed. Owners accepted all of the above procedures by signing a consent form.

### 2.2. Products and randomization

The animals were randomly and equally distributed (20 dogs per group) into one of the two following groups:

- the control group had no water additive added to the drinking water during the study.
- the Aquadent group received Vet Aquadent^®^ FR3SH water additive (Virbac, Poland), diluted in drinking water (1 mL of water additive per 100 mL of drinking water), every day.

The randomization list was obtained using the randomization program Research Randomizer by blocks of two to have an equal repartition of dogs with similar body weights in both groups. One coordinator allocated the dog in one specific group according to the randomization list but the rest of the team conducting the clinical study (procedures and scoring) were blinded concerning the treatment received.

All dogs received the same diet fed dry (Hill’s® Pet Nutrition Maintenance diet) for the duration of the study and any dental hygiene other than the one tested (dental chews, brushing, toothpaste, etc) was prohibited, in agreement with the owners. Water with or without the additive was offered to dogs *ad libitum*.

### 2.3. Design

The dogs received the tested product cited above (or none) for 30 days. On day 0, all animals had a general physical examination including visual assessment, heart and lungs auscultation, capillary refill time assessment, body weight measurement, general body condition score, and body temperature measurements. Intravenous catheters were placed and blood collected for immediate laboratory work. Urine samples were collected by cystocentesis and immediately evaluated in a UA analyzer to measure pH and specific gravity, and test for protein, glucose, ketones, and blood to assess suitability for anesthesia and dental procedure. Sedation was performed with the use of medetomidine (Sedator, Eurovet Animal Health B.V, intramuscularly) at recommended doses and butorphanol (Torbugesic, Zoetis, intramuscularly 0.1 mg/kg) followed by pre-oxygenation with an oxygen mask. After sedation was achieved, the animal received propofol (Propomitor, Orion Pharma Poland sp. zoo, intravenously) at recommended doses for induction of general anesthesia, and an endotracheal tube was placed. General anesthesia was maintained using isoflurane (Isoflurin, VetPharma Animal Health SL, inhaled) at recommended dose and oxygen (Tlen medyczny AirLiquid Polska sp. zoo, inhaled) for the duration of the dental procedure. Fluid therapy (intravenous) was introduced with Ringer’s solution (Plyn Ringera Fresenius Kabi Polska sp. zoo) at standard infusion rates (4 mL/kg/h). During anesthesia, cardio-respiratory functions were monitored. A temperature maintenance system was also put in place.

The periodontal disease (PD) status was evaluated under general anesthesia on day 0 and included comprehensive oral health assessment with periodontal probing, dental charting, and full mouth radiography as well as assessment and notification of Gingival bleeding Index. If the PD status was above grade 2, according to the AVDC staging (17), the dog was excluded from the trial.

A professional dental cleaning procedure and teeth polishing were then performed under general anesthesia. Scaling and polishing quality was evaluated by the use of a disclosing solution (IC plaque, IM3) to ensure that all plaque and calculus were removed. If not, the scaling and polishing procedure was repeated.

After dental procedures were done, the animal was allocated to an Intensive Care Unit cage with an oxygen supply and received atipamezole (Atipam, Eurovet Animal Health BV, intramuscularly) in relevant dose to reverse sedation.

On day 30, the veterinarian assessed the animal before sedation and used the same drugs and doses as on day 0 to anesthetize the animal. Oral assessment and all scorings were performed on generally anesthetized animals by one scorer (JG) who is an experienced researcher in the periodontal field and board-certified specialist in veterinary dentistry, performing interrater reliability tests on an annual base (18).

The oral assessment methods for gingivitis, plaque, and calculus were applied to the following 9 (5 + 4) target teeth: Maxilla – I3, C, P3, P4, M1; Mandible – C, P3, P4, and M1. The entire buccal surface of the target teeth on both sides of the mouth was scored for the three parameters. The gingival bleeding index (GBI) (19) was first scored, using a periodontal probe, as follows: 0: no inflammation; 1: mild inflammation (slight change in color, slight edema and no bleeding on probing); 2: moderate inflammation (redness, edema, glazing of surface and delayed bleeding on probing) and 3: severe inflammation (immediate bleeding on probing). A plaque-disclosing solution (IC plaque, IM3) and dental loupes (with light set up to be as close to the daylight as possible) were then used to assess the coverage (0: none; 1: <25%; 2: 25–50%; 3: 50–75%; and 4: 75–100%) and thickness (1: Light; 2: Medium; 3: Heavy) of the plaque on the crown. The plaque score for each tooth was obtained by multiplying the coverage and thickness of plaque as described in previous studies (20, 21). The plaque was brushed from the surface of the teeth prior to the calculus coverage assessment. Finally, the calculus index was evaluated with the use of a dental explorer to assess the coverage of the whole crown (same scoring scale as for the plaque coverage). Each target tooth was assessed and mean mouth scores for GBI, plaque, and calculus indices were calculated for each dog.

### 2.4. Statistics

A Shapiro–Wilk test was used to check the Gaussian distribution of the raw data. Non-parametric tests (Mann–Whitney or Wilcoxon signed-rank tests) or parametric tests (Student’s t-test) were used accordingly for data that did not follow a Gaussian distribution or those that did follow a Gaussian distribution, respectively. The plaque and calculus indices were compared between groups with a Mann–Whitney test. A Student’s t-test was used to compare the evolution of GBI (difference of scores between day 30 to day 0) between groups and a Wilcoxon signed-rank test was used to compare the GBI between time points in each group. A difference was considered significant for a two-tail value of *p* <0.05. The data analysis for this article was generated using the Real Statistics Resource Pack software (Release 8.8 - Copyright 2013–2023, Charles Zaiontz, www.real-statistics.com). Data are presented as median scores (Q1-Q3) for indices at a specific time point or as mean (SD) for the evolution of GBI.

## 3. Results

### 3.1. Plaque and calculus indices

**Figure 1:**
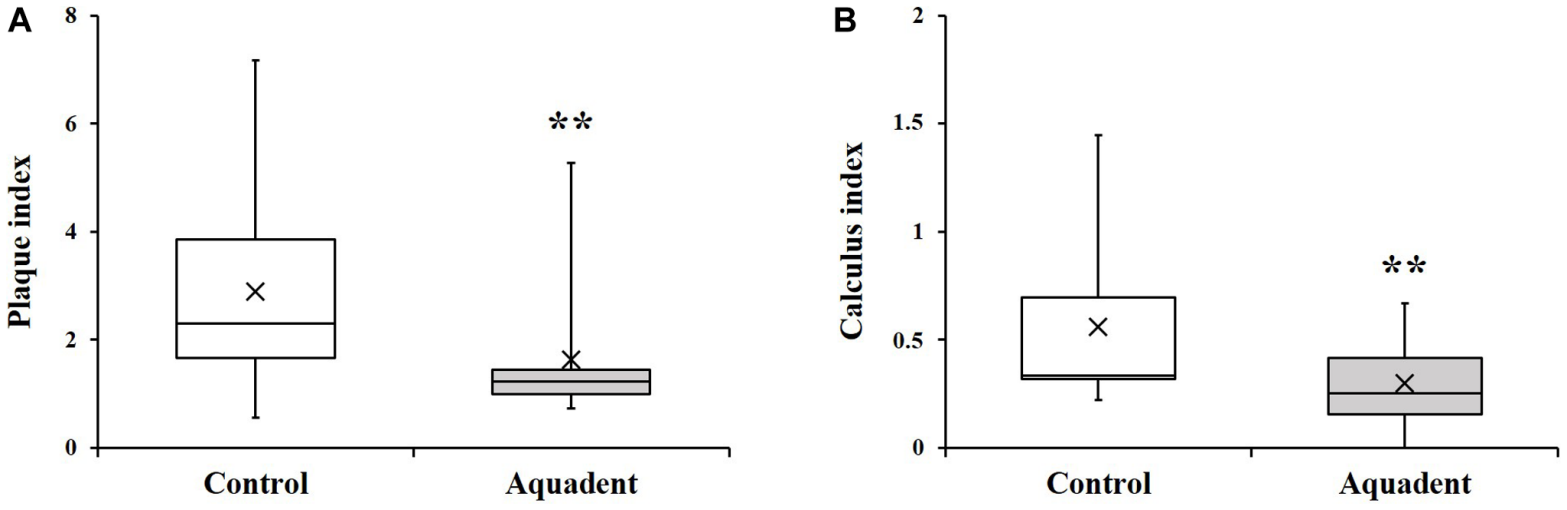
Plaque and calculus indices. Box plot showing the plaque **(A)** and calculus **(B)** indices obtained on day 30. The cross (x) indicates the mean value; **, *p* < 0.01 compared to the control group.

The group of dogs receiving the water additive (Aquadent) for 30 days had significantly lower plaque and calculus indices than the group with no water additive (control group, Figure 1). Indeed, the plaque indices [median (Q1-Q3)] were of 1.22 (0.99–1.44) *vs*. 2.31 (1.65–3.86) in the Aquadent and control groups, respectively (*p* = 0.003, Figure 1A) corresponding to a 47% reduction in median score in the Aquadent group. The calculus indices [median (Q1-Q3)] were of 0.25 (0.15–0.42) *vs*. 0.33 (0.32–0.69), respectively (*p* = 0.005, Figure 1B), corresponding to a 24% reduction in the Aquadent group.

### 3.2. Gingival bleeding index

**Figure 2:**
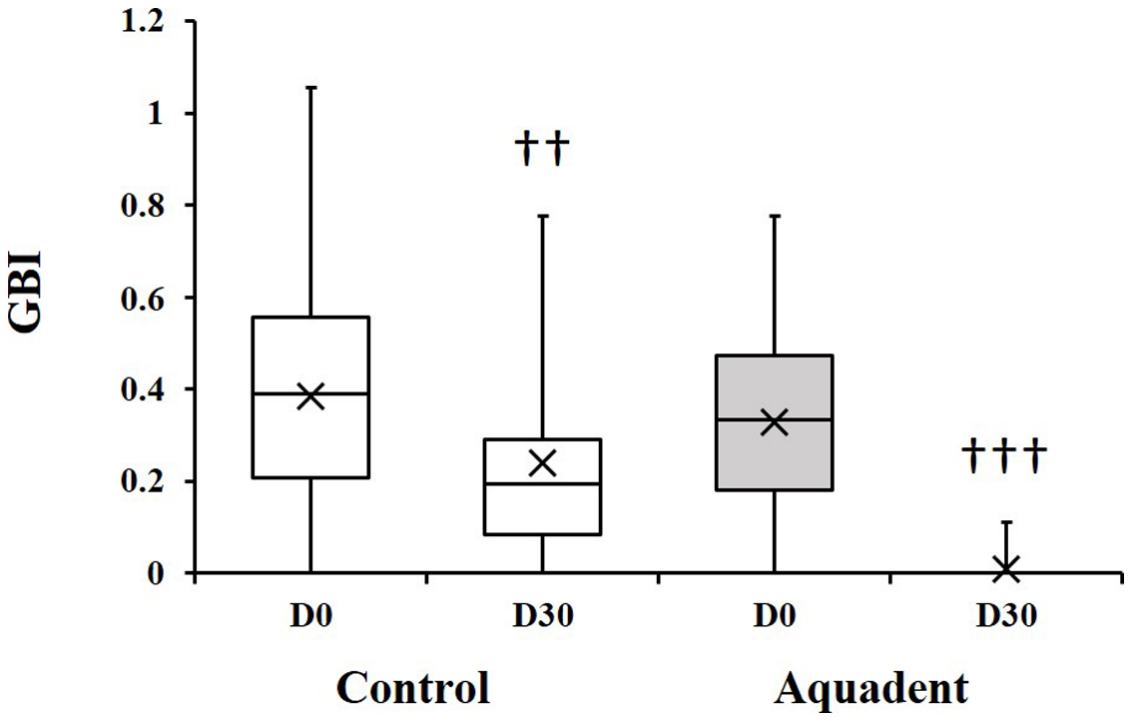
Gingival bleeding index (GBI). Box plot showing the GBI obtained on day 0 (D0) and day 30 (D30). The cross (x) indicates the mean value; ♰♰: *p* = 0.01 and ♰♰♰: *p* = 0.0005 compared to day 0.

The gingival bleeding index (GBI) was assessed on day 0 and day 30 and the evolution of this parameter (difference of scores between day 30 and day 0) calculated for each dog. The GBI significantly decreased in both groups on day 30 compared to day 0. In the control group, this index went from 0.39 (0.21–0.56) to 0.19 (0.08–0.29) (51% reduction, *p* = 0.01), and went from 0.33 (0.18–0.47) to 0.00 (0.00–0.00) in the Aquadent group (100% reduction, *p* = 0.0005, Figure 2). When comparing the evolution of this parameter between groups, the decrease was significantly greater in the group receiving the water additive with a mean (SD) reduction of 0.14 (0.21) and 0.32 (0.23) in the control and Aquadent groups, respectively (*p* = 0.016).

## 4. Discussion

The data presented here show for the first time that the water additive tested (Vet Aquadent^®^ FR3SH^™^) can significantly decrease plaque and calculus accumulation on teeth and can improve the gingival health of dogs when given for 30 days after a professional dental cleaning.

Very few studies have reported the efficacy of water additives in dogs and none have reported a reduction of that magnitude (>40%) on plaque formation (22, 23). It is, indeed, quite challenging to show the clinical efficacy of water additives, as they do not rely on any mechanical effect, unlike other dental care regimens like brushing or dental chews. Different hypotheses can be made in an attempt to explain such efficacy and will be discussed later in the discussion. However, it is important to note first that the water additive is efficient against plaque and calculus accumulation only if a professional dental cleaning has been performed before its use. A former study performed by the same authors on dogs that had not received professional scaling has shown that the water additive was inefficient to remove dental deposits when they were already present on teeth (24). Furthermore, its beneficial effect on gingiva was significant only if associated with tooth brushing (24). Such findings suggest that Aquadent can limit the accumulation of plaque on teeth but cannot remove it and that, to be efficient on the gingiva, plaque must be limited and/or regularly removed (thanks to tooth brushing, for example). Plaque is formed by bacteria on the tooth surface that are organized in biofilms, which make them harder to remove (2). Therefore, mechanical scraping or professional teeth cleaning is usually necessary to remove plaque and calculus (the latter is formed from the mineralization of the plaque) (2). The ability of Aquadent to limit plaque accumulation, as shown in this study, suggests that it may act early on biofilm formation.

This study was performed following evidence that the pomegranate extract present in the water additive could reduce the survival of some canine oral bacterial species involved in plaque formation and periodontal disease (*P. gulaea* and *N. canis*) when tested *in vitro* on strains organized in biofilms (12). The study also showed that the extract could limit their growth in planktonic suspensions (12). The effect of Aquadent on plaque and calculus accumulation after dental scaling in dogs could then be attributed, at least in part, to the control of bacterial growth provided by the pomegranate extract (12). However, other components or parameters may also be involved since the control of bacterial growth by the pomegranate may require a certain contact time. In the *in vitro* study (12), the pomegranate solution was left in contact with the biofilm for 15 min while the dog may be in contact with the water additive for a shorter duration (when the dog drinks). The other ingredients like inulin or erythritol could also play a role in the regulation of plaque accumulation by modulating the bacterial flora, as demonstrated in human or *in vitro* studies (6, 7, 10). The pH of the solution (quite acidic in Aquadent) could also be involved by limiting the proliferation of some bacteria. It is known for example that humans have a lower salivary pH than dogs, less calculus accumulation (25) and that the plaque composition in bacteria is different than the one in dog’s dental plaque (2, 13). It is thought that the difference in salivary pH may explain the difference in plaque composition and formation between humans and dogs (2, 25). One could then think that decreasing the pH of the dog’s saliva and modifying its flora could help reduce the plaque and calculus accumulation in this species. Although the exposure time is reduced with a water additive, a combination of all these properties may explain the effectiveness of Aquadent but further studies would be required to fully understand the mechanism of action.

It has to be noted that the speed and level of plaque accumulation were not assessed in each dog prior to the study start and therefore not taken into account to randomize the dogs. This lack of pre-trial assessment that was done for ethical reasons (it would have required a third general anesthesia) may constitute a bias and a limitation of this study.

Like in other studies (22, 26) and as expected following professional dental cleaning procedure, the GBI was significantly reduced in both groups at day 30. However, the GBI reduction was significantly greater in the Aquadent group than in the control group (reaching 100% reduction based on the median scores in the Aquadent group but only 51% reduction in the control group), suggesting an effect of the Aquadent solution on top of the beneficial effects of scaling and polishing on oral health. In addition to its effects on bacterial growth, pomegranate also has antioxidant properties that are of interest for oral diseases, as demonstrated in other studies (27–29). Such antioxidant and antibacterial properties could explain in part the beneficial effect of Aquadent on the gingiva. A modification of the subgingival microbiota and biofilm, as demonstrated with some mouthrinses in human (30), could be another hypothetical explanation. Targeting the subgingival plaque is indeed of great importance since it is the one that can eventually lead to inflammation and periodontal disease, and it is known that controlling the supragingival plaque alone is ineffective in controlling the progression of PD (2).

These combined properties of the water additive could explain the beneficial effects of Vet Aquadent^®^ FR3SH^™^ on gingival and oral health. However, further research would be required to verify the different hypotheses developed here and fully understand the mechanism of action of this water additive.

## 5. Conclusion

The water additive tested (Vet Aquadent^®^ FR3SH^™^) was shown to maintain oral health in dogs after a professional dental cleaning, by decreasing the accumulation of plaque and calculus on teeth and improving gingival health. Since a water additive is easier to use daily than tooth brushing and is less caloric than dental chews, it could be an interesting option to recommend after a professional dental cleaning, notably to pet owners who are reluctant to brush their dog’s teeth or give a dental chew.

## Data availability statement

The raw data supporting the conclusions of this article will be made available by the authors, without undue reservation.

## Ethics statement

The animal studies were approved by Virbac ethical committee (Approbation numbers: EU-ERC/202205-01). The studies were conducted in accordance with the local legislation and institutional requirements. Written informed consent was obtained from the owners for the participation of their animals in this study.

## Author contributions

JG and CN contributed to the conceptualization and design of the study and wrote the original draft. JG and DZ collected and organized the data. CN analyzed the data. JG, DZ, and CN contributed to the interpretation of the results and manuscript revision. All authors contributed to the article and approved the submitted version.

## Funding

The study was financed by Virbac SA.

## Conflict of interest

CN is a Virbac SA employee and JG received financial support from Virbac SA.

The remaining author declares that the research was conducted in the absence of any commercial or financial relationships that could be construed as a potential conflict of interest.

## Publisher’s note

All claims expressed in this article are solely those of the authors and do not necessarily represent those of their affiliated organizations, or those of the publisher, the editors and the reviewers. Any product that may be evaluated in this article, or claim that may be made by its manufacturer, is not guaranteed or endorsed by the publisher.
